# Fetal alcohol spectrum disorder among pre-adopted and foster children

**DOI:** 10.1186/s12887-020-02164-z

**Published:** 2020-06-03

**Authors:** Ariel Tenenbaum, Asaf Mandel, Talia Dor, Alon Sapir, Orly Sapir-Bodnaro, Pnina Hertz, Isaiah D. Wexler

**Affiliations:** Medical Unit for Adoption and Foster Care, Department of Pediatrics, Hadassah University Medical Center, Mount Scopus Campus, 92140 Jerusalem, IL Israel

**Keywords:** Adoption, Alcohol, Congenital anomalies, Fetal alcohol syndrome

## Abstract

**Background:**

Fetal alcohol spectrum disorder (FASD) is a leading cause of neurodevelopmental disorders. Children in foster care or domestically adopted are at greater risk for FASD. The aim of this study was to determine the prevalence or risk for FASD in a selected population of foster and adopted children.

**Methods:**

Children between 2 and 12 years who were candidates for adoption in foster care were evaluated for clinical manifestations and historical features of fetal alcohol spectrum disorder based on established criteria for FASD.

**Results:**

Of the 89 children evaluated, 18 had mothers with a confirmed history of alcohol consumption during pregnancy. Two children had fetal alcohol syndrome and one had partial fetal alcohol syndrome. In addition, five had alcohol-related neurodevelopmental disorder, one had alcohol-related birth defects, and a single child had manifestations of both. Of the 71 children in which fetal alcohol exposure could not be confirmed, many had manifestations that would have established a diagnosis of FASD were a history of maternal alcohol consumption obtained.

**Conclusions:**

In a population of high-risk children seen in an adoption clinic, many had manifestations associated with FASD especially where prenatal alcohol exposure was established. The reported prevalence in this study is higher than that reported in our previous study of younger children. This is most likely due to the higher number of children diagnosed with alcohol-related neurodevelopmental disorders that typically manifest at an older age.

## Background

Fetal Alcohol Spectrum Disorder (FASD) is a leading cause of acquired developmental delay in the world [1]. FASD results from the teratogenic effects of alcohol on the developing fetus, especially during the first trimester [2]. The manifestations associated with FASD are heterogeneous including highly specific dysmorphia (e.g. smooth philtrum, thin vermillion border of the upper lip, small palpebral fissures, and head circumference ≤ 10th percentile), prenatal and/or postnatal growth deficiency, other types of non-specific birth defects, neurodevelopmental abnormalities, and neuroanatomic defects [3–5]. The effects of exposure to alcohol on the fetus are long term and associated with developmental disabilities, cognitive impairments, psychiatric disorders, and social maladjustment [4–6]. The diagnosis of FASD is important for patients and their families since early intervention, appropriate medical care, and social support are essential for improving outcome and quality of life [7], including better adjustment in school, enhanced interaction with peers, employability, and enhanced social integration.

FASD is highly prevalent in many countries for which statistics are available. It is estimated that the prevalence of FASD in populations of younger school children may be as high as 2–5% in the US and some Western European countries [8]. The incidence is particularly high in children in orphanages and foster care despite the fact that the diagnosis is often missed [9–13].

It is often difficult to diagnose FASD and the diagnosis may be missed especially among children who are institutionalized or adopted [11, 14]. In Israel, the number of documented children carrying the diagnosis of FASD is extremely low. Senecky et al. reported that during a ten year period (1998–2007), only 6 patients were listed with a diagnosis of FASD in two of the four Israeli health maintenance organizations that provide universal health coverage for Israeli citizens. This is despite the fact that significant maternal consumption of alcohol during pregnancy has been documented among Israeli women [15].

In a previous study, we found that among 100 children seen at an adoption clinic, 15% of the children under the age of 2 years met the criteria for FASD or would meet the criteria for a diagnosis of FASD at an older age if neurocognitive disabilities or certain dysmorphic characteristics were to become recognizable [14]. These findings suggested that there is an increased prevalence of FASD in selected populations [10]. Reasons for possible under-diagnosis of FASD could be related to lack of awareness among health professionals regarding the diagnostic features of FASD, absence of systematic data collection, lack of universally accepted diagnostic criteria for the diagnosis of FAS and FASD, or unawareness of the importance of listing FASD as a diagnostic entity [14–16].

Many of the features of FASD especially those related to neurodevelopment are diagnosed later in life when behavioral abnormalities and learning disabilities become more evident. We believe that our previous study in which only very young children were investigated underestimated the prevalence of FASD among adopted or foster children in Israel. We therefore conducted a study of older children aged 2–12 years among children who are candidates for adoption or in foster care to determine whether there was an increase in the proportion of children fulfilling the diagnostic criteria of FASD. This population was selected because adopted and foster children are at greater risk for having the manifestations of FASD and the availability of a cohort that is drawn from a national sample.

## Methods

Eighty-nine children between the ages of 2–12 years referred to the Hadassah Mount Scopus Medical Adoption Unit for medical and developmental assessments were evaluated. The adoption unit has a contractual agreement with the government of Israel to evaluate children born in Israel who are in foster care and are potential candidates for adoption. The study participants were from all population sectors, socioeconomic status levels, and geographic areas representative of the general population of Israeli foster children in this age group.

Diagnostic criteria for FASD and clinical manifestations associated with FASD were based on the US Institute of Medicine’s (IOM) diagnostic classification with modifications introduced by a Canadian Task Force and Hoyme et al. [17–19]. All of the children in the study were evaluated by two pediatricians (IW and AT) who assessed for signs of dysmorphology associated with FASD utilizing the lip/philtrum guide of Astley and Clarren [19] and uses a ruler to measure palpebral fissure length. For assessment of maternal alcohol consumption, biographical information on the parent(s) including a history of alcohol consumption during pregnancy prepared by the child’s case worker and a review of all the children’s medical and social records including developmental and social assessments. With regard to neurobehavioral deficits, many cases, children had already undergone a recent formal developmental assessment at a child development center. If one had not been performed, a developmental psychologist working in our clinic performed the assessment and prepared a written report. It should be noted that in the older Hoyme et al. criteria, the diagnosis of neurocognitive deficits were more “gestalt” without specific cutoffs or required number of domains impacted. Information collected during the evaluation was recorded in the patient’s medical record and subsequently collated. The study was approved by the Hadassah University Medical Center Institutional Review Board.

For many of the patients, complete or reliable information was not available for various reasons, including lack of cooperation of the biological parents in situations in which children were removed from the home due to neglect or abuse, children who were found abandoned, or unreliability of the information obtained (e.g., lack of external validation related to ethanol consumption). This is a particularly significant problem associated with this patient population because most of the categories of FASD require a confirmed maternal exposure to alcohol.

With reference to maternal alcohol consumption, we followed the recommendations of the Canadian Task Force that a history of alcohol consumption during pregnancy was sufficient. Our study population presented unique problems with regard to prenatal alcohol exposure. The preponderance of children in our study who were placed in foster care or became candidates for adoption because of the unavailability of biological parents (abandoned children, parents incapable of raising children because of intellectual disability, psychiatric disorders, or serious substance abuse) or parents accused of abuse in which admitting to alcohol usage would damage their legal standing. As such, in many cases it was difficult to obtain a reliable history of prenatal alcohol exposure. Due to policies of Israel’s Child Services Agency, we could not contact biological parents. We adopted a conservative approach to maternal alcohol consumption. Unless the information was reliable and confirmed by an outside source (social worker, family), we did not categorize the mother as having consumed alcohol during pregnancy.

It should be noted that Hoyme and colleagues have published updated guidelines for the diagnosis of FAS and FASD [20] and categorization of prenatal alcohol exposure. However, our study was initiated before 2015, and we utilized the existing criteria available at that time. As part of the discussion, the impact of the updated criteria as defined by Hoyme et al. as it relates to the diagnosed cases of FASD in our study will be discussed.

Since the alcohol consumption history of many mothers was unknown, we classified children with the clinical signs of ARND or ARBD without a known maternal history as being at risk. That is, if a maternal history of alcohol consumption is eventually obtained, then these children would meet the criteria for a diagnosis of either ARND or ARBD based on the existing clinical manifestations.

## Results

Demographic data for children and patients are shown in Table 1. For 20.2% of the mothers, there was a confirmed history of alcohol consumption during pregnancy and for 4.5%, consumption of both alcohol and illicit drugs. Some of the mothers were involved in high-risk behaviors such as concomitant drug use or had a partner consuming drugs and alcohol (Table 1).

**Table 1:** Demographic characteristics of patients and biological parents

Patient’s age (years)	
Mean (SD)	5.5 ± 2.24
Median	5.5
Range	2.16–11
Age distribution (n)	
2 y	13
3–6 y	53
7–12 y	23
**Gender**	
Males	50
Females	39
**Religion of parents**	
Jewish	62
Moslem	2
Christian	0
Mixed	6
Unknown	19
**Maternal history**	
Alcohol use	18
Drug use	8
Both	4
Promiscuity	24
**Paternal history**	
Alcohol use	3
Drug use	7
Both	1

The age range for the children in our study population was 2.16–11.0 years (median 5.5, mean 2.24 ± 5.5) and the demographic factors including age distribution are shown in Table 1. Table 2 presents the clinical finding for both children with confirmed maternal alcohol consumption and those whose mothers either denied alcohol consumption during pregnancy or the history was unknown. Of the children with documented maternal exposure to alcohol during pregnancy, two children fulfilled the criteria for FAS while a third had facial dysmorphology but lacked the other characteristics (e.g., small head circumference for gestational age) of FAS. Seven children had neurological findings and two had birth defects. In terms of diagnostic categorization, besides the two patients with FAS, one patient had partial FAS, five had ARND, one had ARBD, and a single patient had both ARND and ARBD. In summary, 3 children fit the diagnostic criteria for the umbrella diagnosis of FASD, 3 had neurocognitive manifestations associated with FASD but did not fulfill all of the criteria for ARND, and 5 patients who had no manifestations associated with FASD.

**Table 2:** Clinical characteristics of patients^a^

	***Confirmed maternal alcohol consumption*** ***(n = 18)***	***Alcohol consumption denied or unknown*** ***(n = 71)***
Mean Age (y)	4.6 ± 1.5	5.7 ± 2.4
No abnormalities	4	21
Facial dysmorphology	4	3
Low birth weight (≤ 10%)	2	1
Failure to thrive^b^	6	13
Head circumference ≤ 10%	2	1
Neurocognitive/neurodevelopmental	7	39^c^
Birth defects	2	4

^a^Several children had more than one characteristic

^b^ Height and/or weight ≤ 10th percentile

^c^Three children had neurocognitive signs but were not formally diagnosed with a neurocognitive disorder

Of the children in which maternal exposure to alcohol during pregnancy could not be documented, three children had facial dysmorphology but none of these patients had all the diagnostic criteria for FAS were a history of fetal alcohol exposure to be obtained. A very large number of children without reported alcohol exposure had clinical features such as failure to thrive defined as being less than 10% for weight and length, neurological findings, and birth defects. The frequency of neurocognitive/ neurodevelopmental manifestations was 63%. Only 13 out of 71 children (18.3%) in this group had no clinical manifestations that could be associated with FASD.

## Discussion

In a group of children evaluated in a national medical adoption unit, 20.2% of the patients had in utero exposure to alcohol (Table 1). Most likely, this is an underestimation as information regarding alcohol exposure during pregnancy was not available for many of the mothers. Among the children with known exposure to alcohol, nearly half met the criteria for a diagnosis of FASD (including FASD, partial FASD, ARBD and ARND). Of the children for whom a history of alcohol exposure could not be obtained, many would fit the criteria for FASD if information became available concerning maternal consumption of alcohol.

Comparing the current study to our previous study on children aged 0–2 years [14], the diagnosed prevalence of FASD increased with age. This is most likely due to the fact that ARND is difficult to diagnose during the first years of life. This is especially true for neurocognitive, behavioral and social adaptation disorders which are usually diagnosed in the context of an educational framework. In fact, it is likely that we also underestimated the incidence as it can be challenging to diagnose subtle learning disabilities and attention deficit in children aged 2–5 y.

In 2016, Hoyme and colleagues published revised criteria for the diagnosis of FASD [20]. For all categories of FASD, there was a requirement for neurobehavioral impairment with or without cognitive impairment. According to the new criteria, a diagnosis of ARND cannot be made before the age of 3, and more rigid standards for the diagnosis of cognitive and behavioral impairments were established which included the number of cognitive domains impacted. However, this group did relax the inclusion criteria for the diagnosis of neurobehavioral impairment by establishing a cutoff of − 1.5 S.D. in place of − 2.0 S.D. that are utilized in other published criteria. As pointed out by Viljoen and colleagues when they compared the earlier IOM criteria with the revised in the same cohort, the new criteria resulted in fewer children receiving a diagnosis of FAS and FASD. These authors discuss the clinical and social implication related to the application of the new criteria [21]. In terms of our study, we may have diagnosed fewer children with ARND as we used the older criteria.

The high rate of both FASD and the risk for developing FASD is in line with research from other countries. FASD features were found in more than half of Russian orphans living in baby homes in Murmansk, Russia [22]. A similar prevalence was found among adopted children from Eastern Europe [9]. Moreover, previous studies have also found that children with FASD are overrepresented in foster care and adoption [11–13]. For example, 50% of the surveyed children with FASD or FAS in Washington State had at least one adoptive parent and 15.4% had foster parents [10].

The current study adds to recent reports suggesting an increase in alcohol abuse in Israel, especially among teenagers and young adults which is consistent with international trends. This is reflected in statistics regarding the increasing abuse of alcohol in the general population [23], alcohol level in fatal casualties in motor vehicle accidents [24], and the number of children brought to the emergency room with alcohol poisoning [25].

Our study raises two questions. First, is there any intervention possible for women who are at high-risk for prenatal alcohol exposure such as in our study group. Here the answer is somewhat pessimistic as there are many psychosocial factors associated with these mothers such as psychiatric illnesses, drug abuse, poverty, etc. What could potentially improve the situation is early identification of women to either provide them with familial planning or in the case of women already pregnant, intense social interventions geared to curb drinking behavior. The second question is the applicability of our study to the general population. The high rate of prenatal alcohol exposure and the number of children diagnosed or at risk for FASD should raise the question of whether the diagnosis of FASD is often overlooked among Israeli children [11]. One reason might be the under-reporting of alcohol usage during pregnancy. Weiss et al. found that among 2477 Israeli women who gave birth in one medical center between the years 1999–2000, only 1.13% reported drinking alcohol in small amounts and low frequency during pregnancy [26]. The researchers of the study questioned their results in comparison to similar studies that found higher prevalence. It is likely that in this study there was underreporting due to fear from stigmatization, denial and/or reluctance to share this information. The more recent study done by the Israel Anti-Drug Authority in 2010 lends support to this assumption as it was found that out of 3815 women who gave birth in 3 different hospitals, 17.1% reported drinking alcohol during pregnancy and 0.8% drank excessively at least once in the first trimester [27]. Another possibility for under-diagnosis is the lack of awareness of healthcare professionals regarding the prevalence of maternal drinking and the diverse manifestations of FASD [15]. Quite often, the history of maternal alcohol consumption is not available to mental health professionals who are evaluating children for developmental or learning disabilities.

In general, real-world diagnosis of FASD remains challenging for the clinician for several reasons. First, there is still no unified agreed upon criteria for the diagnosis. Second, many of the updated criteria for diagnosing FASD especially ARND have become more complicated and require extensive cognitive and behavioral testing. Often this requires referral to a specialized center with expertise in FASD which is often an option not available to many families. Third, in many cases, information related maternal consumption of alcohol during pregnancy is either unavailable or unreliable. Fourth, FASD is often a disorder in evolution, and as we have shown in this study, the prevalence of FASD increases with age since many of the clinical manifestations related to dysmorphism and neurocognitive/neurobehavior only become apparent at older ages. Fifth, when assessing neurocognitive /neurobehavioral status, it is often challenging to assess the impact of social environmental conditions and adversity on cognitive development.

A limitation particular to this study is the fact that there were significant psychosocial factors that could contribute to neurocognitive and neurobehavioral challenges displayed by these subjects. This remains a general challenge associated with the diagnosis of ARND, as few, if any, neurologic manifestations are specific for prenatal alcohol exposure [28, 29]. In the old criteria which were later revised, there was no threshold for the amount of alcohol [17]. Even when there is a history of maternal alcohol consumption that is quantifiable, there is always a question of whether a particular manifestation is related to the toxic effects of alcohol or some other factor. In any case, it is important for clinicians and mental health specialists to be aware that maternal alcohol consumption may have been contributory.

Despite these limitations, it is still important to identify children who have a very high chance of having FASD so that they can receive the appropriate intervention. Diagnosis of FASD at an early stage is beneficial as early intervention may minimize many of the cognitive, behavioral, and social problems associated with FASD [7]. It is also extremely important to identify mothers with a history of ethanol consumption as this will facilitate early diagnosis and possibly prevent future cases in the same family [6, 7]. However, because of the stigma which is often linked to children of FASD and their parents, it is important to develop approaches that are supportive and encourage families to bring their children for evaluation.

## Conclusions

This study shows that there is a high rate of FASD and risk for developing FASD in a selected population of adopted or foster children. This study confirms previous studies indicating that FASD had been previously underdiagnosed in this high-risk group. Children above the age of 2 y fitting the criteria for FASD rises as neurodevelopmental and behavioral assessments are more accurate. The findings of our study emphasize the importance of being aware of FASD especially given the prevalence of maternal consumption of alcohol during pregnancy. As intervention is important and potentially beneficial, it would be important to identify children with FASD or at risk for developing FASD at an early stage.

## Data Availability

The datasets used and/or analyzed during the current study are available from the corresponding author on reasonable request with personal identifying information removed.

## Abbreviations

ARBD: Alcohol-related birth defects
ARND: Alcohol-related neurodevelopmental disorders
FAS: Fetal alcohol syndrome
FASD: Fetal alcohol spectrum disorder
